# Transgender Noninclusive Healthcare and Delaying Care Because of Fear: Connections to General Health and Mental Health Among Transgender Adults

**DOI:** 10.1089/trgh.2016.0024

**Published:** 2017-02-01

**Authors:** Kristie L. Seelman, Matthew J.P. Colón-Diaz, Rebecca H. LeCroix, Marik Xavier-Brier, Leonardo Kattari

**Affiliations:** ^1^ School of Social Work, Georgia State University, Atlanta, Georgia.; ^2^ Department of Sociology, Georgia State University, Atlanta, Georgia.; ^3^ Department of Psychology, Georgia State University, Atlanta, Georgia.; ^4^ Colorado Department of Public Health & Environment, Denver, Colorado.

**Keywords:** discrimination, health, healthcare, mental health, minority stress model, transgender

## Abstract

**Purpose:** There are many barriers to reliable healthcare for transgender people that often contribute to delaying or avoiding needed medical care. Yet, few studies have examined whether noninclusive healthcare and delaying needed medical care because of fear of discrimination are associated with poorer health among transgender adults. This study aims to address these gaps in the knowledge base.

**Methods:** This study analyzed secondary data from a statewide survey of 417 transgender adults in the Rocky Mountain region of the United States. Independent variables included noninclusive healthcare from a primary care provider (PCP) and delay of needed medical care because of fear of discrimination. Dependent variables assessed general health and mental health.

**Results:** Transgender individuals who delayed healthcare because of fear of discrimination had worse general health in the past month than those who did not delay or delayed care for other reasons (*B*=−0.26, *p*<0.05); they also had 3.08 greater odds of having current depression, 3.81 greater odds of a past year suicide attempt, and 2.93 greater odds of past year suicidal ideation (*p*<0.001). After controlling for delayed care because of fear of discrimination, having a noninclusive PCP was not significantly associated with either general health or mental health.

**Conclusion:** This study suggests a significant association between delaying healthcare because of fear of discrimination and worse general and mental health among transgender adults. These relationships remain significant even when controlling for provider noninclusivity, suggesting that fear of discrimination and consequent delay of care are at the forefront of health challenges for transgender adults. The lack of statistical significance for noninclusive healthcare may be related to the measurement approach used; future research is needed to develop an improved tool for measuring transgender noninclusive healthcare.

## Introduction

Transgender and gender nonconforming people frequently experience exclusion and discrimination in healthcare settings.^1–3^ Yet, they may need to seek medical attention in pursuit of gender-affirming hormones and surgical procedure(s)^4–7^ in addition to other health or mental health conditions. Although in recent years there has been a growing amount of research documenting barriers to reliable healthcare for this population^1,2,8^ and a lack of transgender competence among health and mental health providers,^8,9^ few studies have quantitatively assessed the relationship between delaying healthcare because of fear of discrimination, noninclusive healthcare, and mental or physical health in this population. This study aims to address these gaps in the research.

In this article, the term *transgender* refers to individuals who identify their gender as different from or incongruent with their assigned sex at birth. We sometimes use the term transgender alongside of the word *gender nonconforming*, which includes people who may or may not identify with the word transgender but whose gender may be nonbinary and/or different from the man/woman or masculine/feminine gender binary of predominant U.S. culture.

In addition, as this article focuses on noninclusivity among healthcare providers, we define *transgender noninclusive healthcare* as occurring when a healthcare provider demonstrates a lack of competence, attention, and/or initiative in adequately providing medical treatment to and affirming the identities of transgender patients according to the best available science. Furthermore, we conceptualize *delaying healthcare because of fear of discrimination* as any incident in which transgender individuals avoid seeking professional medical care when needed because of anticipated mistreatment from healthcare providers, medical staff, and/or other patients.

## Barriers to Effective Healthcare for Transgender People

For transgender people, healthcare services are rife with issues of discrimination and noninclusive care from doctors, clinicians, and staff. Discrimination consists of unequal treatment compared with patients who are not transgender; we consider it to be an example of noninclusivity, a broader construct focused on whether a provider is integrating best practices in work with transgender patients.

Transgender individuals are often forced to navigate a healthcare system that is resistant at best and at times openly hostile toward transgender people's needs.^10^ Previous research has found that healthcare was the most common setting in which transgender individuals experienced discrimination compared with other settings such as housing and employment.^8^ However, knowledge about transgender health and specific needs for the community continues to be an inadequately resourced and crucial area for study.^8,11–13^ Results from the Virginia Transgender Initiative Health study, which surveyed 350 transgender individuals, indicated a severe lack of culturally competent and transgender-friendly staff and providers.^8^

Even when transgender patients have regular primary care providers (PCPs), not being able to disclose their transgender identity to healthcare providers was associated with increased odds of discrimination and overall lack of care.^8^ The odds of discrimination were even higher among those in lower socioeconomic brackets. In one study, those who had sought care for hormone therapy, transgender-related surgery, or gynecological care were more likely to experience discrimination than those who did not reveal their transgender status or did not seek medical intervention for physically transitioning to their gender identity.^1,8^

The decision to disclose one's gender identity and sex assigned at birth to healthcare and other service providers is complex. Transgender individuals can find it difficult to balance expressing their gender identity with the fear of being a target for violence or discrimination in healthcare and other services.^14^ Although being recognized and addressed as the gender with which they identify is an essential issue for transgender people,^15^ medical providers and clinic staff often persist in using incorrect pronouns (i.e., misgendering) or challenging the individual's gender identity.

Experiencing noninclusive healthcare, including discrimination, affects the quality of life and help-seeking behaviors of transgender patients. In the largest national survey to date of transgender discrimination in healthcare in the United States (*N*=6450 respondents), researchers found that 28% of respondents reported postponing needed medical care because of fear of discrimination.^2^ In the same study, 19% reported being refused care because of their gender identity, 28% reported being subjected to verbal harassment, and 2% reported being victims of physical violence in medical settings. Compounding these problems, 50% of respondents indicated that they had to teach their medical provider about basic transgender information.^2^

Another study found that transgender individuals were concerned that if they disclosed their gender identity, service quality might be compromised, either through substandard care, problematic notes placed by providers in their medical records, or discriminatory referral to other medical providers solely on the basis of one's transgender identity.^16^ Considering the evidence regarding the occurrence of discrimination and lack of transgender inclusivity in healthcare, as well as the likelihood that transgender patients are delaying needed medical care because of fear of discrimination, there is a need for research looking at how delaying care may relate to patients' general and mental health.

## Minority Stress Model

Minority stress, which is a conceptual model for understanding high rates of poor mental health in a disadvantaged population, was first conceptualized and applied in a study of urban dwelling gay men in the mid-1990s.^17^ Meyer argues that minority stress is composed of three chronic stressors (internalized homophobia, stigma, and experiences of discrimination and victimization) that are unique to gay people and emerge as a result of living in a heterosexist culture.

In the two decades since first being introduced, the minority stress model has been increasingly adopted across disciplines, with mental health researchers applying, extending, and further conceptualizing the model to fit the unique situations and chronic stressors of various minority or disadvantaged groups.^18–23^ Only recently has the minority stress model been applied to mental health research focusing specifically on transgender individuals.^24–26^

The use of a minority stress model within transgender research contributes to our collective knowledge of both the unique stressors faced by transgender and gender nonconforming individuals and the effects of these stressors on mental and physical health.^27^ The most commonly identified and explored stressor for transgender individuals is discrimination.^27,28^ Discrimination has been explored in a number of different ways, including antitrans discrimination,^28–30^ gender-related discrimination,^31^ and discrimination in public spaces.^26,32^

Reisner et al. further explore discrimination in public spaces and argue that fear of discrimination (*anticipated* stigma) and experienced discrimination (*enacted* stigma) in healthcare lead to a substantial increased risk of adverse emotional and physical health as well as postponing needed and/or preventative healthcare.^32^ Additional stressors include perceived transgender stigma,^33,34^ internalized transphobia,^34,35^ gender-related rejection and victimization,^31^ nonaffirmation of gender identity,^27,36^ and nondisclosure of transgender identity.^34^

Collectively, all of these identified stressors are unique to transgender and gender nonconforming individuals and are associated with increased risk of psychological distress,^33^ suicide ideation and attempts,^2,31,37^ alcohol and drug use,^2,37^ and worse physical health.^38^ Based on this model, we can theorize that experiencing noninclusive healthcare (a form of *enacted stigma*), as well as delaying healthcare because of fear of discrimination (*anticipated stigma*), contributes to stressors for transgender people, manifested as worse mental health and self-reported general health.

## Current Study

Although the literature details the various forms of discrimination that transgender and gender nonconforming people frequently face in accessing healthcare, as well as the likelihood that transgender patients may delay care because of anticipated discrimination, few studies quantitatively examine whether delaying care because of fear of discrimination or experiencing particular forms of noninclusive healthcare are associated with worse mental health and general health for transgender adults. This is striking, considering that past research indicates that discrimination and a lack of transgender-specific knowledge are rampant in healthcare provision and a sizeable portion of this population may be delaying healthcare services because of fear of discrimination.

This study adds to the literature in this area by using a statewide survey of transgender adults in one state in the Rocky Mountain region of the United States to address the gaps in the research about the connection between noninclusive healthcare provision, delaying care because of fear of discrimination, and transgender health.

First, we will descriptively explore the characteristics of transgender individuals who have a noninclusive provider or have delayed care because of fear of discrimination and whether they differ in notable ways from those who have access to an inclusive provider or those who have not delayed care because of fear of discrimination. Then, we use multivariate models to assess whether having a noninclusive PCP and delaying care because of fear of discrimination are associated with worse general health and mental health, after controlling for demographic and other variables that tend to be associated with health, such as having health insurance, exercise frequency, and annual household income. Our two primary hypotheses are as follows:

(1) Having a PCP who is not transgender inclusive (operationalized using a five-item composite scale based on examples of noninclusive PCP care) will be significantly associated with worse general health and mental health among transgender adults.
(2) Delaying needed medical care because of fear of discrimination will be significantly associated with worse general health and mental health among transgender adults.

## Methods

### Procedures

This project involved secondary data analysis of a statewide community-based survey of 417 transgender adults in the Rocky Mountain region of the United States. To participate, individuals had to identify as either transgender or gender nonconforming and reside in Colorado. Survey questions were modeled on the CDC's Behavioral Risk Factor Surveillance System (BRFSS), with input from community organizations and transgender community members.^39^ Some questions were added and others were modified to be more reflective of transgender experiences and identities.

Online respondents were recruited in 2014 through advertisement by LGBT organizations, health providers, colleges and universities, and religious communities. Additional respondents, recruited through local events and conferences or who visited the GLBT Community Center of Colorado's office, completed paper copies of the survey.^39^ Since the current analyses involved only de-identified data, this study was considered “not human subjects research” by the lead author's university and thus did not require IRB approval.

### Measures

Most health conditions were queried through single items rather than composite measures. We selected covariates that were either behaviors that are known to be related to health, such as engagement in exercise, or demographics that are frequently related to health disparities (race/ethnicity and income).

The following covariates were included in our multivariate models: (a) time since last visit to a doctor for a routine checkup (dichotomized as either within the past 2 years or >2 years ago); (b) exercise in the past month (a yes/no dichotomous variable); (c) health insurance coverage (a yes/no dichotomous variable); (d) current annual household income (recoded into $25,000 intervals, up to $75,000 or more); (e) number of adults currently living in the household; (f) number of children currently living in the household; (g) race/ethnicity (recoded as a dichotomous white/person of color variable because of the small number [<20%] of persons of color, with white Hispanic respondents categorized as persons of color); and (h) whether one has ever been told by a health professional that one has a depressive disorder (a yes/no dichotomous variable). The number of adults and children in the household were added as covariates so that current annual household income could be examined while accounting for household size.

We had two predictor variables of interest. First, to assess noninclusive healthcare by a PCP, respondents were first asked whether their PCP (or the provider they see most regularly) provides transgender-inclusive healthcare. Those who responded *No* then were asked to indicate why they feel that their provider is not inclusive; precise examples of noninclusivity were developed through input from transgender community members regarding common indicators that a healthcare provider is not competent with and affirming of transgender patients.

Respondents could select as many items as they wished from a checklist that included (a) not enough knowledge on transgender-related healthcare needs; (b) not comfortable with patients who identify as transgender; (c) does not address my transgender-specific healthcare needs, only other medical needs; (d) office policies and forms are not transgender inclusive; (e) office does not provide a welcoming environment for transgender patients; and (f) other (specify). We then computed a composite score of PCP noninclusiveness, with a score of 1 for each check box (a through e),^*^ whereas those who had earlier indicated that their PCP was inclusive were assigned a score of 0.

We then computed a summed score across the five items so that higher scores indicated *less* inclusive care (see Table 1 for scale descriptive statistics and reliability). Our second predictor variable of interest was a dichotomous item (yes/no) regarding whether participants had ever delayed getting needed medical care in the past 12 months because of fear of discrimination.

**Table 1.** **Variable Descriptive Statistics (*N*=417)**

Continuous variables	Range	*M*	SD	Median	Cronbach's α
Annual household income	1 (<$25,000)–4 (>$75,000)	2.09 (approx. $25,000–$50,000)	1.15	$25,000–$50,000	
No of adults in household	1–7	2.11	1.12	2	
No of children in household	0–7	0.34	0.86	0	
Noninclusive PCP scale	0–5	0.83	1.51	0	0.78
General health	1 (Poor)–5 (Excellent)	3.37	1.04	3 (Good)	

PCP, primary care provider.

Our dependent variables included both continuous and categorical (dichotomous) variables related to health. We used one continuous variable, which was general health (how respondents would rate their overall health on a five-point scale ranging from *Poor* to *Excellent*). The general health variable met the assumptions for linear regression, including having a normal distribution (skew and kurtosis were both <±1). Dichotomous-dependent variables included (a) current depression (yes/no), assessed through eight items from the Patient Health Questionnaire (PHQ-8), which is used in the BRFSS to detect depressive symptoms;^40^ (b) suicide attempt (in the past year); and (c) suicidal ideation (past year).

Items for the PHQ-8 are modified, as they are in the BRFSS,^40^ so that each statement references a 2-week time period, such as *Over the last 2 weeks, how many days have you had little interest or pleasure in doing things?* Respondents indicated a number response between 0 and 14. For each item, responses were scored so that 0–1 days=0, 2–6 days=1, 7–11 days=2, and 12–14 days=3. These scores were summed, and those that were 10 and above were designated as indicating current depression.^40^ In this sample, the PHQ-8's Cronbach's α=0.91, which indicates strong internal reliability.

### Statistical analyses

We used SPSS, version 22, for our statistical analyses. Owing to the value of understanding which subgroups of transgender adults may experience noninclusive care from their PCP (reported by 38.7% of the sample) and/or may delay care because of fear of discrimination (31.1% of the sample), we descriptively explored associations between these two variables and some of the particular demographic and health variables from our models that would be of primary interest to practitioners (Table 2). As many of the health variables are strongly correlated to one another, we explored bivariate associations with noninclusive care and delayed care rather than multivariate models.

**Table 2.** **Demographics and Health of Respondents by Experiences of Noninclusive PCP and Delaying Care Because of Fear of Discrimination in the Past Year**

Demographic and health variables	Overall sample (*N*=417), % (*N*)	Noninclusive PCP, % (*n*)	Transgender inclusive PCP, % (*n*)	*χ*^2^ test result	Delayed care (because of fear of discrimination), % (*n*)	Did not delay care (because of fear of discrimination), % (*n*)	*χ*^2^ test result
Routine checkup (past 2 years)							
Yes	82.4 (333)	71.1 (108)	89.6 (216)	22.22^a^	71.9 (82)	86.7 (221)	11.65^a^
No	17.6 (71)	28.9 (44)	10.4 (25)		28.1 (32)	13.3 (34)	
Annual household income							
<$25K	42.4 (167)	46.8 (66)	38.9 (91)	11.10^b^	48.2 (53)	40.6 (97)	12.18^c^
$25K–$50K	25.9 (102)	29.1 (41)	24.4 (57)		30.9 (34)	24.3 (58)	
$50K–$75K	12.4 (49)	5.7 (8)	17.1 (40)		12.7 (14)	11.3 (27)	
$75K+	19.3 (76)	18.4 (26)	19.7 (46)		8.2 (9)	23.8 (57)	
Race/ethnicity							
White	83.7 (334)	79.6 (117)	85.9 (201)	2.60	77.3 (85)	85.5 (207)	3.65^d^
Person of color	16.3 (65)	20.4 (30)	14.1 (33)		22.7 (25)	14.5 (35)	
General health							
Poor	5.3 (22)	7.8 (12)	4.1 (10)	6.48	8.8 (10)	4.7 (12)	14.85^c^
Fair	13.3 (55)	17.6 (27)	11.5 (28)		21.2 (24)	11 (28)	
Good	34.1 (141)	32.7 (50)	34.4 (84)		36.3 (41)	32.5 (83)	
Very good	33.9 (140)	30.7 (47)	35.7 (87)		26.5 (30)	36.5 (93)	
Excellent	13.3 (55)	11.1 (17)	14.3 (35)		7.1 (8)	15.3 (39)	
Current depression							
Yes	44 (158)	53.7 (72)	37.8 (82)	8.55^c^	67 (67)	37.2 (84)	24.81^a^
No	56 (201)	46.3 (62)	62.2 (135)		33 (33)	62.8 (142)	
Suicide attempt (past year)							
Yes	9.9 (39)	15 (22)	7.2 (17)	5.97^b^	22.3 (25)	5.7 (14)	22.07^a^
No	90.1 (355)	85 (125)	92.8 (219)		77.7 (87)	94.3 (233)	
Suicidal ideation (past year)							
Yes	36 (142)	47.6 (70)	28.8 (68)	13.90^a^	58.9 (66)	27.9 (69)	31.55^a^
No	64 (252)	52.4 (77)	71.2 (168)		41.1 (46)	72.1 (178)	

^a^ *p*<0.001.

^b^ *p*<0.05.

^c^ *p*<0.01.

^d^ *p*>0.10.

A chi-square test of association indicated that having a noninclusive PCP was significantly associated with routine checkup in the past year (the likelihood of getting a checkup was significantly less likely when one's PCP was perceived as not transgender inclusive). In addition, chi-square tests of association indicated that having a noninclusive PCP was related to a greater likelihood of current depression, having a suicide attempt in the past year, and experiencing suicidal ideation in the past year (i.e., mental health was worse among those with a noninclusive provider).

Current annual household income was also associated with having a noninclusive PCP, with *post hoc* analyses with correction for alpha inflation (Bonferroni style) indicating that the proportion of those with income between $50,000 and $75,000 were less likely to have a noninclusive PCP than would be expected by chance.

Those who delayed needed medical care because of fear of discrimination were significantly less likely to have had a routine checkup in the past 2 years, and more likely to be currently depressed or have had suicidal ideation or a suicide attempt in the past year (Table 2). General health and annual household income were also significantly related to delayed care because of fear of discrimination, with *post hoc* analyses with correction for alpha inflation (Bonferroni style) indicating that those who delayed care were less likely to have an income of $75,000 or more, more likely to report their health as *Fair*, and less likely to report their health as *Excellent* than would occur by chance (Table 2) compared with those who did not delay care because of fear of discrimination.

For the multivariate models, multiple linear regression was used for the model with a continuous dependent variable and logistic regression for the models with a dichotomous dependent variable. Correlations for all model variables were examined; none had more than weak correlations with one another (not shown here). Covariates were entered in Block 1, and then the predictor variables of interest (noninclusive PCP scale and delaying care because of fear of discrimination) were added in Block 2.

As some of the predictor variables were missing not at random, dropping cases with missing data would lead to biased estimates^41^; we therefore chose to retain such cases by using multiple imputation to replace missing values, with five imputations utilized. Based on the patterns of missingness in our data, we used the fully conditional specification approach to multiple imputation, also known as chained equations imputation. Any differences in our models before and after imputation are noted in our Results section.

## Results

### Sample

Of the 417 transgender and gender nonconforming Colorado residents who responded to this survey, 30.3% (*n*=119) identified as transgender women, 24.9% (*n*=98) as transgender men, 18.3% (*n*=72) as gender queer/gender fluid, 15% (*n*=59) as women,^†^ 5.6% (*n*=22) as men, 4.6% (*n*=18) as transgender, and 1.3% (*n*=5) as agender/no gender. More than half (52.2%, *n*=216) were assigned a male sex at birth. Most identified as being white (88.4%, *n*=352), 8.8% (*n*=35) identified as multiracial, and 2.9% (*n*=11) identified as other races. Less than 1 in 10 respondents were Hispanic (6.7%, *n*=28). Participants reported their ages by selecting the relevant age range; *25 to 34 years old* was both the median and mode response.

About three out of four participants (75.7%, *n*=296) reported that they had participated in physical activities or exercises other than their regular job in the past month. The majority of respondents (85.7%, *n*=349) had some form of health coverage. The most common types of insurance (some participants had more than one type) were plans obtained through one's own work (33.7%, *n*=117) or someone else's work (21.6%, *n*=75), Medicaid (19.3%, *n*=67), Medicare (10%, *n*=35), and health insurance bought directly by oneself (8.4%, *n*=29) or someone else (5.2%, *n*=18). A small minority (7.2%, *n*=25) received veteran's insurance.

Descriptive statistics for model variables not already detailed are displayed in Table 1. There are relatively high occurrences of mental health conditions in this sample, particularly current depression (44%), depression as ever told by a health provider (63.4%), and anxiety as told by a provider (52.7%).

### Multiple linear regression model

One sequential multiple regression model was constructed to examine self-reported general health. Table 3 includes information about the multiple regression model, with model results based on the original data and coefficient estimates based on the data after multiple imputation. Any differences between the model before and after imputation are noted in a footnote in Table 3.

**Table 3.** **Multiple Linear Regression Model (Using Imputed Data) Predicting General Health**

	General health (*N*=398)	
	Block 1	Block 2
	*B* (s.e.)	*B* (s.e.)
Control variables		
Routine checkup in past 2 years (y/n)	0.01 (0.13)	0.08 (0.13)
Exercise in past month (y/n)	0.50^a^ (0.12)	0.51^a^ (0.12)
Health insurance (y/n)	−0.14 (0.14)	−0.11 (0.14)
Annual household income	0.22^a^ (0.05)	0.20^a^ (0.05)
No. of adults in household	−0.07 (0.05)	−0.07 (0.04)
No. of children in household	0.05 (0.07)	0.05 (0.06)
Race (white/POC)	−.05 (0.13)	<0.01 (0.13)
Depression (ever told by provider)	−0.45^a^ (0.10)	−0.40^a^ (0.10)
Predictor variables		
Noninclusive PCP scale		−0.05 (0.03)
Delay care—fear discrimination		−0.26^b^ (0.12)
Model results (original data only)		
*F* value	9.41^a^	8.61^a^
Adjusted *R*^2^	0.19	0.21

Before imputation, in Blocks 1 and 2 of the general health model, the number of adults in the household was significant at the *p*<0.05 level. Constant not displayed here.

^a^ *p*<0.001.

^b^ *p*<0.05.

POC, person of color.

Each block of the General Health model had an *R* significantly different from 0 (*F*=9.41, *p*<0.001, and *F*=8.61, *p*<0.001 respectively). Control variables accounted for 19% of the variance in self-reported general health; once adding the predictor variables of interest, the full model accounted for 21% of the variance in self-reported general health.

After accounting for control variables, noninclusive PCP care was not statistically significantly associated with self-reported general health among this sample of transgender adults. Delaying healthcare because of fear of discrimination was statistically significantly associated with general health, *B*=−0.26, *p*<0.05. For individuals who reported delaying healthcare because of fear of discrimination, self-reported general health was 0.26 points lower than for transgender individuals who did not delay healthcare or delayed healthcare for other reasons.

In terms of the covariates, exercising in the past month was associated with a self-reported general health score about 0.51 points higher than those who did not exercise in the past month (*p*<0.001). Each step increase in current annual income was associated with a 0.2 point increase in self-reported general health (*p*<0.001). Finally, ever being told by a healthcare provider that one was depressed was associated with a general health score of 0.4 points lower than those who were never told they were depressed (*p*<0.001).

### Logistic regression models

Three sequential logistic regression models were constructed predicting current depression, suicide attempt in the past year, and suicidal ideation in the past year. Any differences between the models before and after imputation are noted in Table 4.

**Table 4.** **Logistic Regression Models (Using Imputed Data) Predicting Current Depression, Suicide Attempts (Past Year), and Suicidal Ideation (Past Year)**

	Current depression (*N*=352)		Suicide attempt (past year) (*N*=385)		Suicidal ideation (past year) (*N*=385)	
	Block 1	Block 2	Block 1	Block 2	Block 1	Block 2
	OR (95% CI)	OR (95% CI)	OR (95% CI)	OR (95% CI)	OR (95% CI)	OR (95% CI)
Control variables						
Routine checkup in past 2 years (y/n)	1.62 (0.82–3.19)	1.21 (0.59–2.48)	2.19^ (0.97–4.96)	1.69 (0.71–4.02)	1.63 (0.89–2.99)	1.23 (0.65–2.35)
Exercise in past month (y/n)	0.27^a^ (0.14–0.51)	0.24^a^ (0.13–0.46)	1.49 (0.65–3.43)	1.40 (0.60–3.30)	0.70 (0.41–1.19)	0.64 (0.37–1.11)
Health insurance (y/n)	1.67 (0.76–3.64)	1.53 (0.69–3.43)	0.97 (0.36–2.59)	0.83 (0.30–2.32)	1.44 (0.73–2.86)	1.29 (0.64–2.62)
Annual household income	0.58^a^ (0.45–0.75)	0.62^b^ (0.47–0.80)	0.65^c^ (0.44–0.97)	0.69^ (0.45–1.07)	0.66^a^ (0.52–0.84)	0.69^b^ (0.54–0.89)
No. of adults in household	1.16 (0.92–1.46)	1.16 (0.92–1.46)	1.09 (0.81–1.47)	1.13 (0.82–1.55)	1.11 (0.91–1.36)	1.11 (0.90–1.37)
No. of children in household	1.41^c^ (1.02–1.94)	1.42^c^ (1.03–1.95)	0.85 (0.45–1.63)	0.83 (0.42–1.65)	1.18 (0.88–1.59)	1.18 (0.87–1.60)
Race (white/POC)	1.48 (0.72–3.01)	1.20 (0.58–2.52)	1.51 (0.60–3.80)	1.27 (0.49–3.26)	2.25^c^ (1.17–4.32)	1.93^d^ (0.96–3.90)
Depression (ever told by provider)	8.14^a^ (4.42–14.98)	7.86^a^ (4.21–14.67)	6.24^b^ (2.08–18.71)	5.59^b^ (1.79–17.44)	4.45^a^ (2.57–7.69)	4.03^a^ (2.28–7.13)
Predictor variables						
Noninclusive PCP scale		1.11 (0.92–1.33)		0.99 (0.80–1.23)		1.10 (0.94–1.28)
Delay care—fear discrimination		3.08^a^ (1.59–5.95)		3.81^a^ (1.78–8.15)		2.93^a^ (1.71–5.02)
Model results (original data)						
Omnibus model test (*χ*^2^)	83.13^a^ (*df*=8)	102.81^a^ (*df*=10)	20.63^b^ (*df*=8)	34.37^a^ (*df*=10)	57.14^a^ (*df*=8)	77.72^a^ (*df*=10)
Nagelkerke *R*^2^	0.36	0.43	0.15	0.24	0.25	.32

Constant not displayed here. The following models had differences in results when only using the original data, compared with the imputed data displayed above: Current depression–number of children in the household was not statistically significantly related to current depression in Block 1 or 2 using the original data. The imputed data may be overstating the importance of number of children in relationship to current depression. Suicidal ideation: in Block 2, exercise in the past month was statistically significant (*p*<0.05) in the original data but did not reach significance using the imputed data; it is possible that the imputed data may be underestimating the influence of this variable in relation to suicidal ideation.

^a^ *p*<0.001.

^b^ *p*<0.01.

^c^ *p*<0.05.

^d^ *p*<0.10.

CI, confidence interval; OR, odds ratio; POC, person of color.

Using the original data, Block 1 of each model was a statistically significant improvement over the constant-only models for all models. Nagelkerke *R*^2^ ranged from 0.15 (suicide attempt) to 0.36 (current depression). Adding the noninclusive provider and delaying care because of fear variables in Block 2 of each model was a statistically significant improvement over each constant-only model, according to the omnibus model test (Table 4). Nagelkerke *R^2^* values ranged from 0.24 (suicide attempt) to 0.43 (current depression).

Having a noninclusive PCP was not significantly related to either current depression, suicide attempt, or suicidal ideation in the past year. Delaying healthcare because of fear of discrimination was statistically significantly positively associated with all outcome variables in the logistic regression models (*p*<0.001). Compared with those who did not delay healthcare or delayed for reasons other than fear of discrimination, those who delayed care because of fear of discrimination had approximately three times greater odds of current depression, almost four times greater odds of a suicidal attempt in the past year, and almost three times greater odds of having suicidal ideation in the past year.

For the covariates, being told by a health provider that one was depressed was statistically significantly associated with current depression, suicide attempts, and suicidal ideation in the past year; after controlling for household size, having a lower annual household income was also associated with greater odds of current depression, suicide attempt (although this was marginally significant after adding the predictor variables of interest in Block 2), and suicidal ideation.

After accounting for noninclusive PCP care and delayed care because of fear of discrimination, those who exercised in the past month had 4.17^‡^ lower odds of current depression than those who did not exercise. Finally, compared with those who were white, people of color had 2.25 greater odds of suicidal ideation in the past year, although this relationship became marginally significant after adding the predictor variables of interest to the model.

## Discussion

Transgender individuals are often forced to navigate a healthcare system that can be unknowledgeable, resistant, and at times hostile toward transgender people's needs.^10^ Understandably, transgender individuals often choose not to seek healthcare because of fear of discrimination.^2^ This study contributes to the literature by using a statewide sample of more than 400 transgender adults to examine the roles of a lack of transgender inclusivity among healthcare providers and delaying care because of fear of discrimination in relation to health indicators among this population.

First, understanding which subgroups of transgender adults report having a noninclusive PCP or delaying care because of fear of discrimination can help with planning interventions to reach these subgroups. As indicated in Table 2, those reporting a noninclusive PCP or who delayed needed medical care because of fear of discrimination were less likely to have had a routine checkup in the past 2 years, and more likely to have current depression, or suicidal ideation or attempt in the past year.

The lack of routine checkups may be driven by the perceptions of lack of inclusive care among PCPs or anticipated discrimination, because PCPs are regularly turned to for such checkups. Thus, there is a crucial need for training for PCPs with regard to providing transgender-inclusive care, as has been recommended by other scholars.^6,9,42^ Providers interested in better serving transgender patients may need to actively seek out those patients (whether or not they are known to be transgender) who are *irregularly* receiving physical examinations and provide communication to all patients about staff persons' expertise and previous training in working with transgender patients and/or the availability of transgender-related care, such as hormone therapy.

In addition, those impacted by noninclusive care or who delay needed medical care because of fear of discrimination trend toward being of lower income and facing mental health struggles; reaching out to these subgroups of transgender adults and connecting them with transgender-inclusive providers is critical. This finding emphasizes the importance of health clinics that reach low-income individuals and have a specialization in transgender healthcare, including mental healthcare.

Those who report delaying needed medical care also were disproportionately represented among those saying their overall health was *Fair* and less represented among those reporting *Excellent* health. On one hand, patients with the most health struggles would be anticipated as believing they “needed medical care” recently, and thus may be more likely to consider delaying medical care than those with excellent health, who might not think they need to see a health provider at all.

Yet, it is concerning that transgender patients whose health is less than good—and who are likely at greatest need for care—are perceiving that health providers will discriminate against them and are, therefore, not seeking out a medical provider. This emphasizes the importance of medical providers communicating their experience, knowledge, and comfort in working with transgender patients to counteract perceptions of anticipated discrimination that may be discouraging patients from seeking care.

The multivariate findings unequivocally supported our second hypothesis: there was a significant association between delaying needed healthcare in the past year because of fear of discrimination and worse general health and mental health (current depression, suicidal ideation, and suicide attempts). Interesting, these relationships were still significant even when controlling for factors of noninclusivity among PCPs, which suggests that one's perception of *possible* discrimination and consequent delay of care are at the forefront of health challenges for transgender adults.

However, it is worth noting that although the noninclusivity items focused on one's PCP (or provider seen most regularly), the delay of needed healthcare variable was framed in reference to the broader category of “needed medical care,” which opens the possibility that some respondents may have had PCPs who were “inclusive” but delayed medical care with another provider because of fear of discrimination, or vice versa. As the delaying care because of fear of discrimination variable was in relation to any needed medical care, it encompasses the possibility that some transgender patients may delay care for transgender-related healthcare needs, whereas others delay seeking care for other medical issues not related to one's transgender identity.

It is important to note that for this population, patients may be forced to seek out several physicians alongside having a PCP because a patient's PCP may or may not be assisting with transgender-specific health needs. This is a significant concern because needing multiple physicians to receive care increases the possibility of encountering healthcare professionals who are not transgender inclusive.

Our study found that transgender patients' general health was significantly related to fear of discrimination from a medical provider, indicating a connection between anticipated discrimination and one's overall (physical and mental) health; although we did not explicitly explore physical health conditions, such as chronic diseases or high blood pressure, this finding regarding general health suggests that there may be some differences in health conditions beyond mental health that are associated with anticipated discrimination.

Fear of discrimination was also significantly associated with poor mental health, in the form of current depression, suicidal ideation, and suicide attempts. Mental health is a crucial dimension of overall health, and the mental health needs of transgender individuals are substantial.^18,43,44^ Our findings corroborate previous research about mental health disparities, as our sample had notably high rates of depression, suicidal ideation, suicide attempts, and anxiety compared to general adult population data for the state of Colorado.^39^ If transgender patients are fearful of experiencing stigma and discrimination, then they are less likely to seek out any sort of care, which can negatively affect their overall health and well-being, including mental health.

In general, we did not find evidence to support our first hypothesis: the noninclusive PCP scale was not significantly associated with any of the health items after controlling for delaying care because of fear of discrimination. The scale utilized in this study focuses only on PCPs, and it is possible that for the types of outcomes we are examining, the inclusiveness of the PCP is not the primary contributor to these specific stressors. In fact, there is a high probability that these patients are seeking out other healthcare providers for care, such as for mental health treatment related to depression, anxiety, or post-traumatic stress disorder.^45^

Further, the noninclusive PCP items and scale have not been psychometrically tested, and the use of yes/no check boxes to indicate examples of noninclusive practices is not as strong as if such items were measured on a continuous scale. Thus, this survey's approach to measuring noninclusivity may affect our ability to detect relationships between noninclusive PCP care and transgender health. These are some factors that may explain why this hypothesis was not supported in this sample.

These limitations are important to emphasize, as notable previous research and best practices guidelines emphasize the importance of transgender-inclusive care for the best health outcomes among transgender and gender nonconforming patients.^1,3,6,15,32^ The lack of significance of this variable in this study should not be interpreted as a contradiction to such guidance, but rather a function of measurement limitations as well as the inclusion of delaying care because of fear of discrimination within the multivariate models.

### Limitations

This study used a one-time survey of a convenience sample of transgender adults in one state. As such, results are likely biased by the lack of random sampling and we cannot interpret causality in the relationship between provider noninclusivity, delaying care because of fear of discrimination, and health. The majority of the sample was white (88%), and only 7% were Hispanic. However, U.S. Census Bureau data indicate that Colorado has a Hispanic population of 21.2%, suggesting that the Hispanic transgender population may have been under sampled.^46^ Furthermore, few older adults participated in this survey. Older transgender adults may not be as connected to community-based LGBT organizations, which was one method used to recruit these participants.

Our approach did not include an analysis of gender identity subgroups, so we could not compare experiences between transgender men versus transgender women, for example. The survey had notable measurement limitations, including those previously discussed in relation to PCP noninclusivity; in addition, the measure of delaying healthcare because of fear of discrimination did not specify what type of discrimination the respondent feared (i.e., whether it was discrimination based on gender identity or some other individual characteristic, such as race or ethnicity).

Finally, the survey coordinators designed this project to include people who identify as transgender or gender nonconforming. Some individuals who are gender nonconforming might not actually use the term *transgender* for themselves, and thus combining both of these groups may hide some distinctions between them.

### Implications

Several implications can be derived from our findings in relation to public health practice and policy. One of the most striking findings suggests that fear of discrimination plays an important role for transgender patients when seeking healthcare services. Specifically, this fear of discrimination and the consequent delay of needed medical care are associated with poorer general health and mental health among this sample of transgender adults, a finding that adds to minority stress research^2,31,38^ about the role of anticipated discrimination for transgender individuals and negative physical and mental health outcomes.

To counteract fears of discrimination, providers need to consistently and clearly communicate the measures they have in place to affirm transgender health and competently serve this population, such as nondiscrimination policies, intake forms that sensitively ask for gender identity, training and accountability for transgender inclusivity among providers, and expertise in transgender healthcare. Inclusivity training should happen not only with providers but also with all members of the healthcare staff, including front desk and administrative staff. We believe it is imperative that providers are cognizant that patients fear discriminatory behavior from their first interaction with staff, for example, using proper pronouns and names, asking for identification, and completing forms.^47–49^

Grant et al. specifically recommend that the medical establishment fully integrates transgender-sensitive care into its professional standards, and this be part of a broader commitment to cultural competency around race, class, and age.^2^ Discrimination and stigma are significant factors for the transgender community when seeking healthcare services, and teaching PCPs and medical staff how to be more inclusive, knowledgeable, and transgender friendly is crucial in alleviating the marginalization the community faces. Taking tangible and visible steps to reach this population and increase provider competence can build trust, which is likely to help encourage transgender patients not to delay seeking medical care when needed.

Next steps for public health research could consist in replicating this survey with a national sample to test regional and state differences. Additional work could be done to conduct psychometric testing of noninclusive healthcare scale, as the scale used in this study has not been thoroughly psychometrically tested. There would also be great value in conducting longitudinal studies of transgender health that examine the connection between provider inclusivity, delaying care because of fear of discrimination, and experiences of discrimination over the life course.

The general health and mental health of transgender adults are affected by many factors beyond a provider's level of transgender inclusivity or delaying healthcare because of fear of discrimination, including stigma encountered across multiple sectors of life. Future research needs to continue to examine the many contributing factors to transgender well-being, beyond and in addition to healthcare settings.

## Notes

^*^ When including the response option *Other* in calculating this scale, Cronbach's α was 0.55, which is a weak level of internal reliability. After dropping the *Other* item from the calculation, Cronbach's α improved to 0.78.

^†^ Each person who participated in the survey had to identify as transgender or gender nonconforming. However, some individuals within the transgender community identify their primary identity as being either men or women after a transition; these subgroups are captured here.

^‡^ This statistic is calculated by using the inverse odds ratio: 1/0.24=4.17.

## Abbreviations Used

BRFSS: Behavioral Risk Factor Surveillance System
PCP: primary care provider
PHQ-8: Patient Health Questionnaire
